# Enzyme Replacement in a Human Model of Mucopolysaccharidosis IVA *In Vitro* and Its Biodistribution in the Cartilage of Wild Type Mice

**DOI:** 10.1371/journal.pone.0012194

**Published:** 2010-08-16

**Authors:** Melita Dvorak-Ewell, Dan Wendt, Chuck Hague, Terri Christianson, Vish Koppaka, Danielle Crippen, Emil Kakkis, Michel Vellard

**Affiliations:** 1 BioMarin Pharmaceutical Inc., Novato, California, United States of America; 2 Buck Institute, Novato, California, United States of America; National Institutes of Health, United States of America

## Abstract

Mucopolysaccharidosis IVA (MPS IVA; Morquio A syndrome) is a lysosomal storage disorder caused by deficiency of N-acetylgalactosamine-6-sulfatase (GALNS), an enzyme that degrades keratan sulfate (KS). Currently no therapy for MPS IVA is available. We produced recombinant human (rh)GALNS as a potential enzyme replacement therapy for MPS IVA. Chinese hamster ovary cells stably overexpressing GALNS and sulfatase modifying factor-1 were used to produce active (∼2 U/mg) and pure (≥97%) rhGALNS. The recombinant enzyme was phosphorylated and was dose-dependently taken up by mannose-6-phosphate receptor (K_uptake_ = 2.5 nM), thereby restoring enzyme activity in MPS IVA fibroblasts. In the absence of an animal model with a skeletal phenotype, we established chondrocytes isolated from two MPS IVA patients as a disease model *in vitro*. MPS IVA chondrocyte GALNS activity was not detectable and the cells exhibited KS storage up to 11-fold higher than unaffected chondrocytes. MPS IVA chondrocytes internalized rhGALNS into lysosomes, resulting in normalization of enzyme activity and decrease in KS storage. rhGALNS treatment also modulated gene expression, increasing expression of chondrogenic genes *Collagen II, Collagen X*, *Aggrecan* and *Sox9* and decreasing abnormal expression of *Collagen I*. Intravenous administration of rhGALNS resulted in biodistribution throughout all layers of the heart valve and the entire thickness of the growth plate in wild-type mice. We show that enzyme replacement therapy with recombinant human GALNS results in clearance of keratan sulfate accumulation, and that such treatment ameliorates aberrant gene expression in human chondrocytes in vitro. Penetration of the therapeutic enzyme throughout poorly vascularized, but clinically relevant tissues, including growth plate cartilage and heart valve, as well as macrophages and hepatocytes in wild-type mouse, further supports development of rhGALNS as enzyme replacement therapy for MPS IVA.

## Introduction

Mucopolysaccharidosis IVA (MPS IVA; Morquio A syndrome; OMIM #253000), is an autosomal recessive disorder caused by deficiency of N-acetylgalactosamine-6-sulfatase (GALNS) [1]. This enzyme hydrolyses sulfate ester bonds and is required during the sequential degradation of the glycosaminoglycans keratan sulfate (KS) and chondroitin-6-sulfate in the lysosomes [2]. Deficiency in GALNS leads to lysosomal accumulation of glycosaminoglycans and subsequent cellular pathology, most notably in connective tissues rich in KS, including cartilage, cornea and heart valve [1], [3]. A number of other cell types, including macrophages [4], [5] and coronary intimal smooth muscle cells [5] also contain lysosomal storage, suggesting that the pathophysiology of MPS IVA may extend beyond the KS-rich tissues. Interestingly, growth plate KS does not accumulate in mouse, in the absence of GALNS, rendering the mouse models inappropriate [6].

Like other MPS disorders, MPS IVA is clinically heterogeneous, ranging from severe skeletal dysplasia with early mortality to milder forms [7]. The skeletal dysplasia is particularly evident in epiphyseal growth plates and vertebrae, and is hallmarked by spondyloepiphyseal dysplasia with genu valga, pectus carinatum, spinal kyphosis and odontoid hypoplasia. Thoracic cage deformity contributes to severe respiratory restriction and potentially pulmonary failure. Odontoid hypoplasia is frequent and, along with ligamentous laxity, often results in cervical instability and high cervical spinal cord compression [8]. Degeneration of articular cartilage leads to early-onset osteoarthritis. Corneal clouding as well as aortic and mitral valve pathology secondary to stenosis are also observed [9]. Unlike most MPS diseases, MPS IVA is not characterized by impairment of mental status.

Growth plate chondrocyte pathology in MPS IVA is characterized by vacuolar distention, defective differentiation, chaotic arrangement and poorly calcified matrix [10], [11]. On the contrary, bone cells, osteoblasts and osteoclasts, appear unaffected [10], [11], [12], although vacuolar distention has been observed in osteocytes [13]. Histological studies confirm that although the bone tissue from MPS IVA patients is reduced in quantity, at the histological and ultrastructural level it is qualitatively comparable to unaffected bone [11], [14]. Anderson *et al* concludes that the cause of dwarfism lies primarily in the deficit in chondrocyte differentiation, rather than abnormal bone formation [10]. Articular cartilage chondrocytes are also vacuolated, disorganized, and exhibit an altered expression of extracellular matrix components [15], changes that may be associated with early-onset osteoarthritis observed in MPS IVA [14]. Cartilage and heart valve spongiosa, the major therapeutic target tissues in MPS IVA, are largely avascular [16], [17], and are as such challenging to penetrate with therapeutic compounds.

Treatment options for children with MPS IVA are limited to bone marrow transplantation and frequent orthopedic surgeries. Patients show incomplete response to bone marrow transplantation, which is furthermore associated with high morbidity and mortality [18]. The advent of enzyme replacement therapy (ERT) brought significant improvement in the management of lysosomal storage diseases, including MPS I, II, and VI, Gaucher disease, Fabry disease and Pompe disease [19]. We here report production and characterization of recombinant human GALNS (rhGALNS) for potential enzyme replacement therapy of MPS IVA. We furthermore describe establishment of a novel model of disease, primary human MPS IVA chondrocytes *in vitro*. In this model we show rhGALNS uptake by lysosomes, subsequent clearance of KS storage and changes in cellular function, in terms of gene expression. Finally, we address the issue of rhGALNS delivery to clinically relevant tissues, and show, for the first time, penetration of the therapeutic enzyme throughout the growth plate, all layers of the heart valve as well as liver macrophages in wild-type mice.

## Results and Discussion

### Production, purification and characterization of rhGALNS

**Figure 1:**
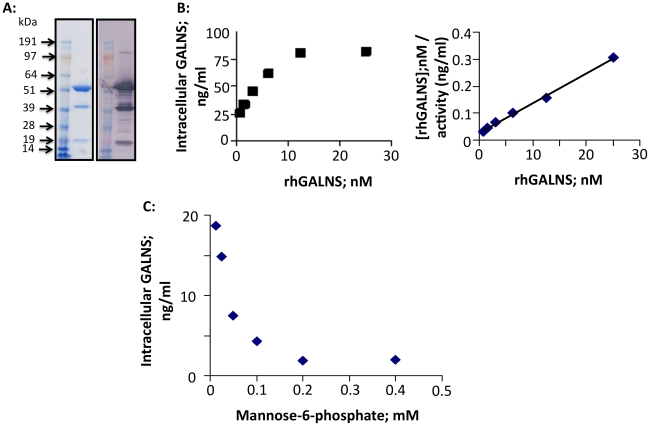
rhGALNS production and characterization. **A:** SDS-PAGE of final purified rhGALNS was stained with Coomassie blue (left) or immunoblotted for GALNS (right) under reducing conditions. **B:** Dose-dependent uptake of rhGALNS (0.78 – 25 nM) by primary MPS IVA fibroblasts (left). K_uptake_  = 2.5 nM was determined by Hanes-Woolf plot linear regression analysis (right). Y axis is reported as [rhGALNS], (nM)/activity (ng/ml), where rhGALNS is the substrate (of the Mannose-6-phosphate (M6P) receptor), and ng/ml represents ng of active enzyme per ml, based on a standard reference preparation of purified rhGALNS with specific activity of 2 U/mg. **C:** M6P inhibited uptake of 2.5 nM GALNS by MPS IVA fibroblasts. Means of 3 independent experiments are presented.

We produced rhGALNS from conditioned media from CHO cells stably overexpressing GALNS and sulfatase modifying factor 1 (SUMF1). SUMF1 encodes the formylglycine-generating enzyme required for activation of all sulfatases including those associated with the mucopolysaccharidoses [20], [21], [22]. The overall purification recovery of rhGALNS was ∼56% with typical specific enzyme activity of 2 U/mg. The enzyme was ≥97% pure based on reverse-phase HPLC. **Figure 1A** shows SDS-PAGE of the purified enzyme, with a major species of ∼55 kDa, and minor ∼40 kDa and ∼19 kDa species under reducing conditions, as described previously [23]. In agreement with previous reports, chromatography confirmed rhGALNS associates as a non-covalent dimer in solution [23]. The enzyme was stable in serum with an extrapolated t_1/2_ of ∼200 hrs, at pH = 7.4 *in vitro*. rhGALNS also exhibited affinity for hydroxyapatite, the major mineral constituent of bone, comparable to osteopontin, a hydroxyapatite-binding protein, and arylsulfatase B (ASB), which is in use as ERT for another MPS disorder with significant skeletal dysplasia, MPS VI (Maroteaux-Lamy Syndrome). On the other hand, α-glucosidase exhibited no affinity to hydroxyapatite (**Figure S1A**).

Mannose-6-phosphate (M6P) residues on the oligosaccharides of lysosomal enzymes are critical to successful ERT, so we evaluated the structure of the oligosaccharides on rhGALNS. [24]. There are two consensus N-linked oligosaccharide sites in the polypeptide sequence of GALNS, Asn204 and Asn423. Mass Spectrometry analysis revealed that all phosphorylated oligosaccharides reside at Asn204, consistent with previous reports [25]. rhGALNS oligosaccharide profile (**Figure S1B**) indicates phosphorylated oligosaccharides make up ∼50% of the total oligosaccharides. Therefore, the molar ratio of phosphorylated oligosaccharide to rhGALNS protein is approximately 1∶1, suggesting that each molecule has the capacity to be taken up by the M6P receptor and delivered to lysosomes, especially since rhGALNS dimerizes in solution.

Addition of rhGALNS to MPS IVA fibroblasts corrected their enzyme deficiency dose-dependently, with a K_uptake_ of 2.5 nM (**Figure 1B**). rhGALNS uptake was M6P receptor-dependent, as it was significantly inhibited by M6P (**Figure 1C**).

### Primary human MPS IVA chondrocytes as an MPS IVA model

**Figure 2:**
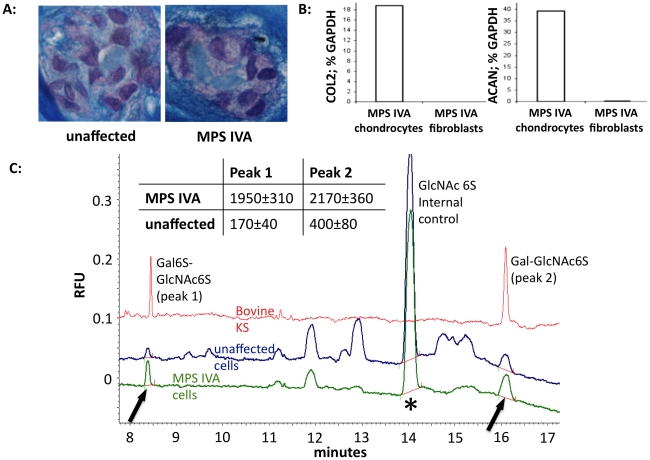
Primary chondrocytes as a model of MPS IVA *in vitro*. **A:** Alcian blue staining of proteoglycans in 6-week cultures. **B:** Collagen 2 (COL2) and Aggrecan (ACAN) qRT-PCR of RNA isolated from 2-week alginate MPS IVA chondrocyte cultures or monolayers of MPS IVA fibroblast cultures. Means of triplicates ±SEM are shown (p<0.01 in both graphs). **C:** Capillary electrophoresis of total cell lysates from 6-week alginate cultures of MPS IVA cells, digested with keratanase II. Keratan sulfate peaks (Area under the curve; AUC) were normalized for internal control and also protein content in cell lysates, as cell numbers in MPS IVA cultures were significantly lower than in unaffected controls. Table shows quantification of peaks expressed as Relative fluorescence units (RFUs)/internal control/µg protein. Experiment was performed in duplicate and representative traces shown.

Murine models lacking active GALNS do not successfully recapitulate the human skeletal disorder, as unlike the patients, they do not exhibit skeletal deformities, but do suffer from substrate accumulation in the brain [26], [27], [28]. The authors ascribe this lack of phenotype to the fact that mice do not produce the same type of skeletal KS as humans [6]. Dermal fibroblasts from patients are also not optimal since they do not exhibit KS accumulation nor pathology [12]. Using primary chondrocytes isolated from iliac crest biopsies of two MPS IVA patients we established a model of human MPS IVA *in vitro*. Primary human chondrocytes, isolated from healthy articular knee cartilage, as well as MPS IVA chondrocytes supplemented with rhGALNS were used as controls. Chondrocytes cultured in monolayers de-differentiate, losing their ability to produce chondrocyte markers, such as *Collagen II* and *Aggrecan* [29]. We maintained chondrocytes in alginate suspension cultures supplemented with IGF-1, TGFβ, transferrin, insulin and ascorbic acid, conditions which support chondrocyte differentiation [29], [30], [31] and extracellular matrix and KS production in culture [32], [33]. We observed chondrogenic differentiation, hallmarked by synthesis of extracellular matrix (**Figure 2A**) and production of major chondrogenic markers *Collagen II* and *Aggrecan* mRNAs (**Figure 2B**). However, this approach also slowed cell growth (**Figure S2**) and limited the material available for study.

We studied MPS IVA cells during proliferating (monolayers, 6-week alginate cultures) and differentiated (15 week cultures) phases. MPS IVA cells retained the fundamental and causative feature of the MPS IVA phenotype in culture, as they had undetectable GALNS activity compared to unaffected cells (**Table 1**). Lysates of long-term MPS IVA cultures were analyzed by Capillary Electrophoresis (CE), which revealed an up to 11-fold increase in KS in MPS IVA cultures, in comparison to unaffected cells (**Figure 2C**), indicating KS accumulation.

**Table 1:** GALNS activity in MPS IVA cells.

	GALNS activity^e^			
	MPS IVA chondrocytes		Unaffected chondrocytes	
nM rhGALNS:	0 nM	10 nM	0 nM	10 nM
Monolayers, 3 days culture^a^	ND^c^	2.2±0.18^c^	0.1±0.003	2.2±0.03
Alginate, 15 weeks culture^b^	ND^c^	1.5^c^	0.3	1.6
Alginate, 3 weeks culture^b^	ND^d^	0.8^d^	0.21	0.7

ND (none detected)  =  values <0.1;

a n = 3;

b n = 1;

c MPS IVA patient 1;

d MPS IVA patient 2.

e Enzyme activity was converted to and reported as ng of active enzyme per ml, based on a standard reference preparation of purified rhGALNS (2 U/mg specific activity). Activity was normalized per µg of total protein present in the cell lysates.

Although the diffusion-limiting properties of cartilage are not recapitulated in cultured chondrocytes, the absence of active rhGALNS and subsequent accumulation of KS, as well as expression of chondrogenic genes, make MPS IVA chondrocytes a valuable new model of human MPS IVA *in vitro*. Translatability of this model to a clinical context *in vivo* will become more apparent with further studies of tissues and cells isolated from MPS IVA patients.

### rhGALNS restores enzyme activity and clears KS in MPS IVA chondrocytes

**Figure 3:**
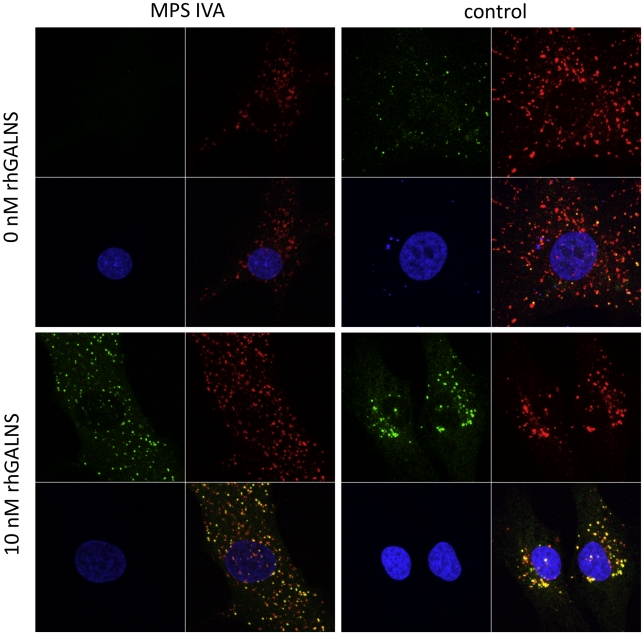
rhGALNS is internalized into lysosomes of MPS IVA chondrocytes. Chondrocytes treated with 10 nM rhGALNS for 3 days exhibited GALNS staining (green), which colocalized (orange) with a lysosomal marker, LAMP1 (red). Confocal images were acquired with identical parameters.

We restored GALNS activity in monolayers and alginate suspension cultures of MPS IVA cells (**Table 1**). rhGALNS trafficked to lysosomes, evident by its colocalization with Lysosomal Associated Membrane Protein-1 (LAMP1) (**Figure 3**). Treatment with 1 nM and 10 nM rhGALNS resulted in dose-dependent uptake of the enzyme in alginate cultures, evident by immunofluorescence (**Figure S3A**). The enzyme was taken up throughout the culture period (**Figure S3B**).

**Figure 4:**
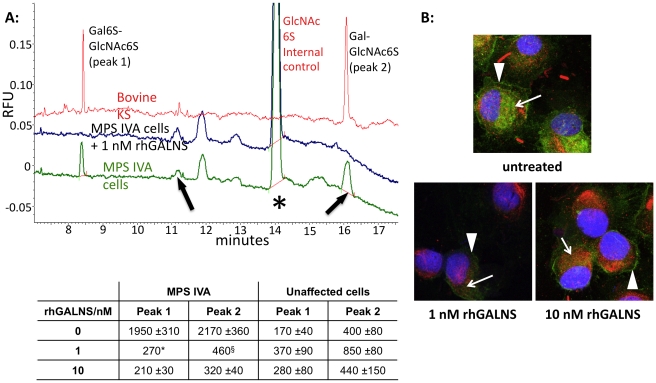
rhGALNS clears KS accumulation from MPS IVA chondrocytes. **A:** Capillary electrophoresis of keratanase II-digested total cell lysates from 6-week alginate cultures treated with rhGALNS continuously. Keratan sulfate peaks (Area under the curve; AUC) were normalized for internal control and also protein content in cell lysates, as cell numbers in MPS IVA cultures were significantly lower than in unaffected controls. Table shows quantification of peaks expressed as Relative fluorescence units (RFUs)/internal control/µg protein. ^§^n = 1 is shown as the second sample had undetectable peaks. Experiment was performed in duplicate and representative traces shown. Green  =  MPS IVA chondrocytes; Blue  =  MPS IVA chondrocytes treated with 1 nM rhGALNS; Red  =  bovine corneal KS standard. Arrows: disaccharide peaks Gal6S-GlcNAc6S and Gal-GlcNAc6S. Asterisk: GlcNAc6S internal control. **B:** KS immunofluorescence in MPS IVA chondrocytes from patient 2 after continuous treatment with rhGALNS. Fresh enzyme was added to culture medium twice a week for 6 weeks. Arrowheads  =  extracellular KS; Arrows  =  intracellular KS. Images were acquired with identical parameters. KS  =  green, LAMP1  =  red, colocalization  =  orange.

In order to assess rhGALNS as viable therapy, we addressed its efficacy in terms of clearing KS accumulation *in vitro*. Long-term MPS IVA cultures that exhibited KS accumulation in comparison to unaffected cells, showed significantly reduced accumulation of KS (∼80 – 100%) when cultured with 1 nM or 10 nM rhGALNS, in cells from both patients (**Figure 4A, B** and **Figure S4**). We did not observe dose-dependence in these experiments, indicating that 1 nM rhGALNS may be sufficient for restoration of enzyme activity. KS accumulation was also cleared from mature MPS IVA cells, which had first accumulated KS for 6 weeks prior to incubation with rhGALNS (**Figure S5**). rhGALNS decreased KS immunofluorescence most significantly in the lysosomal compartment, whereas the immunofluorescence in extracellular matrix was still visible in treated cells from both patients (**Figure 4B** and **Figure S4**). This observation supports that rhGALNS acts in the lysosomal compartment, as the therapeutic enzyme is inactive at extracellular pH and cannot degrade KS without participation of other lysosomal enzymes. Nevertheless, extracellular KS may also be affected, secondary to amelioration in KS turnover and trafficking defects, in response to lysosomal clearance by rhGALNS.

In parallel experiments, superphysiological levels of GALNS in unaffected chondrocytes incubated with 10 nM rhGALNS did not result in changes in KS levels (**Figure 4A** and **Figure S6**), which are efficiently metabolized by the endogenously-expressed enzyme.

### rhGALNS affects function of MPS IVA cells

**Figure 5:**
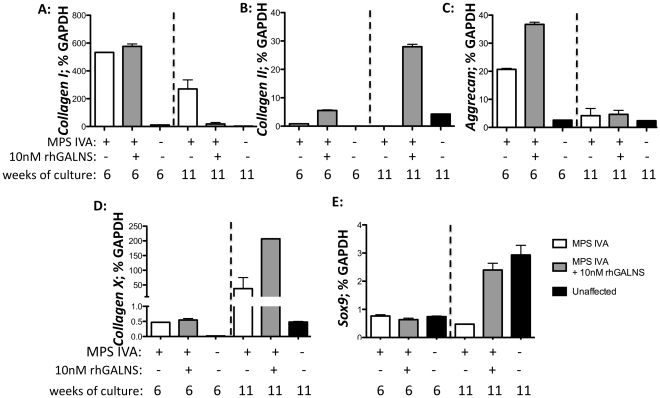
Gene expression changes in MPS IVA chondrocytes after rhGALNS treatment. cDNAs were generated from RNA from 6-week cultures (patient 1) and 11-week cultures (patient 1 and patient 2) of chondrocytes. Cells were either grown in absence (white bars) or presence of 10 nM GALNS (grey bars). Chondrocytes were supplemented with 10 nM rhGALNS throughout culture (6-week cultures) or for the last 5 weeks of culture (11-week cultures). Fresh enzyme was added to culture medium twice a week. Results from unaffected articular adult chondrocytes cells are shown (black bars). Results were normalized for GAPDH expression and shown as means of triplicates ±SEM for patient 1 (6 week cultures) or patient 1 and patient 2 (11-week cultures).

Few studies have specifically addressed the effects of KS accumulation on chondrocyte function and pathophysiology of MPS IVA. De Franceschi and colleagues recently described increased expression of Collagen I and decreased expression of Collagen II and Aggrecan proteins and/or mRNAs in articular cartilage of patients with Morquio syndrome, in comparison to unaffected controls [15]. In agreement with the patient findings, we observed a significantly increased expression of *Collagen I* in both proliferating (6 week) and differentiated (11 week) cultures (**Figure 5A**) and a decreased expression of *Collagen II* in differentiated cultures (**Figure 5B**). In contrast to articular cartilage from patients [15], *Aggrecan* was increased in proliferating MPS IVA chondrocytes, in comparison to unaffected cells (**Figure 5C**). It is possible that the disparate findings in aggrecan expression may result from age and anatomical differences in tissue sources from which the cells were isolated. MPS IVA chondrocytes were isolated from iliac crest growth plates of affected children, whereas control cells were obtained from articular cartilage of a 70-year old adult. This is supported by a higher expression of *Collagen X*, a gene specifically expressed in growth plate hypertrophic chondrocytes, as in MPS IVA cells (**Figure 5D**) [34].

Our results indicate that MPS IVA chondrocytes have undergone phenotypic changes, secondary to lack of GALNS or accumulation of KS, which result in aberrant cellular function, here observed in terms of altered gene expression profile. Bank and colleagues have observed that chondrocytes from two MPS IVA patients process collagen fibrils differently [14]. Altered expression of *Collagen I* and *II* may also indicate a decrease in chondrocyte differentiation, which is hallmarked by high expression of collagen II and low expression of collagen I. This hypothesis is supported by decreased expression of *Sox 9* (**Figure 5E**), a key chondrogenic transcription factor, in differentiating chondrocytes. Alternatively, the increase in *Collagen I* expression in MPS IVA chondrocytes could represent a compensatory mechanism resulting from *Collagen II* reduction, as has been observed in some patients with achondrogenesis type II [35]. Collagen II is the major structural protein that provides cartilage with strength and resilience. Its decreased expression has been associated with “type II collagenopathies”, which range in severity and are hallmarked by dwarfism, skeletal dysplasia, premature osteoarthritis and hearing loss, similarly to MPS IVA. The decrease in *Collagen II* observed in MPS IVA chondrocytes may therefore result in cartilage properties that engender or exacerbate dwarfism, as well as spinal deformity, since collagen II plays a role in formation of nucleus pulposus of the intervertebral disc [36].

rhGALNS treatment stimulated an increase in production of key cartilaginous markers, including *Sox 9* (differentiated cells, **Figure 5E**), *Collagen II* (**Figure 5B**), *Collagen X* (differentiated cells, **Figure 5D**) and *Aggrecan* (proliferating cells, **Figure 5C**). On the other hand, abnormal *Collagen I* expression is decreased by rhGALNS in differentiated cells (**Figure 5A**). To our knowledge this is the first report to describe corrective effect of rhGALNS on function of human MPS IVA chondrocytes. Altered expression of major cartilaginous ECM proteins could lead to alteration in the biomechanical properties of the tissue. ECM perturbations in articular cartilage from two MPS IVA patients were recently characterized by Bank *et al*, who proposed the altered collagen fibrils and proteoglycan arrangements result in cartilage more prone to degeneration [14]. Correction of ECM protein expression in MPS IVA patients by rhGALNS treatment may therefore lead to improvement of biomechanical properties of cartilage resulting in improved joint health and spinal deformities.

### rhGALNS is taken up by therapeutically-relevant tissues *in vivo*

In the present study we studied enzyme biodistribution particularly focusing on clinically-relevant cartilage, heart valve and macrophages, in wild-type mice after multiple enzyme administrations. To avoid non-specific signal associated with antibody detection techniques, we directly visualized exogenously-applied enzyme, by detecting the conjugated Alexa-488 fluorophore. Cellular uptake and subcellular localization of the enzyme was not affected by the fluorophore conjugation (**Figure S7**), making rhGALNS-A488 a suitable tool to study rhGALNS tissue biodistribution *in vivo*. Nonetheless, as one fluorophore represented one monomer of rhGALNS, this method may have resulted in enzyme underestimation.

Delivery of therapeutic enzymes to growth plate chondrocytes is exacerbated by the avascular nature of cartilage [16]. This is also true for the deep layers of the heart valve, which are rich in glycosaminoglycans, but are not well vascularized [17]. Tomatsu and colleagues have shown that a single administration of enzyme does not result in significant delivery to the growth plate [37]. Chronic treatment of GALNS-null mice on the other hand did result in increased enzyme activity in preparations of whole bones, although these studies did not specifically address enzyme biodistribution in cartilage [38], [39]. In these studies Tomatsu and colleagues proposed that rhGALNS tagged with an N-terminus hexaglutamate sequence (E6-GALNS) would target mineralized bone, which contains high amounts of hydroxyapatite. Nevertheless, a study showing delivery of the therapeutic enzyme to the non-mineralized cartilage is still outstanding. We here present the first report of rhGALNS biodistribution specifically in the mouse growth plate and articular cartilage, indicating that non-tagged rhGALNS successfully diffuses through the avascular cartilage to reach chondrocytes, the primary target cells.

**Figure 6:**
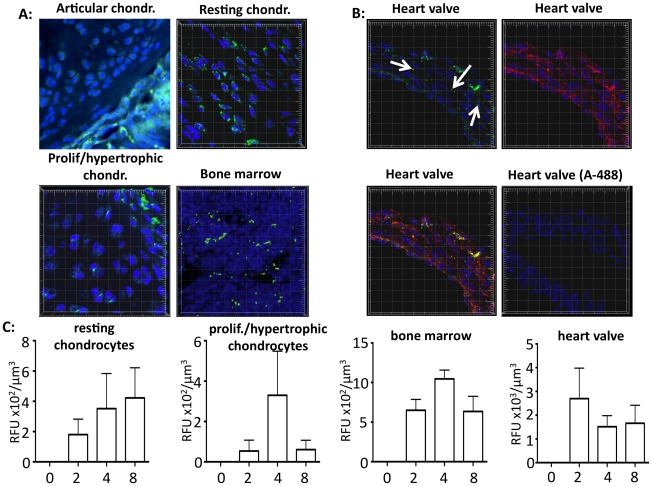
rhGALNS biodistribution in wild-type mouse cartilage, bone marrow and heart valve. **A:** Confocal microscopy of direct rhGALNS-A488 fluorescence in growth plate cartilage, articular cartilage and bone marrow cells (green). Blue  =  DAPI nuclear staining. Original magnification 80x. **B:** rhGALNS-A488 (green) biodistribution throughout the heart valve and colocalization (orange) with lysosomal marker LAMP1 (red). Control section from a mouse treated with the fluorophore A-488/PBS alone is shown. Original magnification 40x. **C:** Confocal stacks are analyzed for fluorescence intensity and data presented as average fluorescent signal (RFU)/µm^3^. Measurements of sections from mice that were either injected with A-488/PBS (0), or rhGALNS-A488 and sacrificed at 2 hr (2), 4 hr (4), or 8 hr (8) after injection (n = 3). Means ±SEM are shown.

Repeat injections of rhGALNS-A488 in mice resulted in enzyme delivery throughout growth plate and articular cartilage (**Figure 6A, D**). Notably, rhGALNS-A488 fluorescence was present throughout the growth plate, albeit in a gradient fashion, with the highest abundance at the cartilage/bone interface, in resting and hypertrophic chondrocytes. Such biodistribution may be a reflection of the proximity of these cells to the vasculature present in the neighboring bone. In favor of this hypothesis is the finding of significant enzyme delivery in the well-vascularized bone marrow (**Figure 6A, D**). Our results demonstrate that rhGALNS is compatible with diffusion through proteoglycan-rich matrices, indicating that increasing the concentration and/or duration of administration of the therapeutic enzyme may result in further improvements in biodistribution.

KS accumulates in the heart valve of MPS IVA patients, and represents a cause of morbidity in this disease [40]. We observed rhGALNS-A488 in the septum, atrium, and heart valve, and we focused on the heart valve for volumetric analysis. Although the heart valve is poorly vascularized [17], we observed significant penetration of rhGALNS-A488, past the endothelium and throughout the valve (**Figure 6B**). Interestingly, enzyme penetration in the heart valve was significantly higher than in growth plate (**Figure 6D**). Lysosomal localization of rhGALNS-A488 is shown in **Figure 6B**.

**Figure 7:**
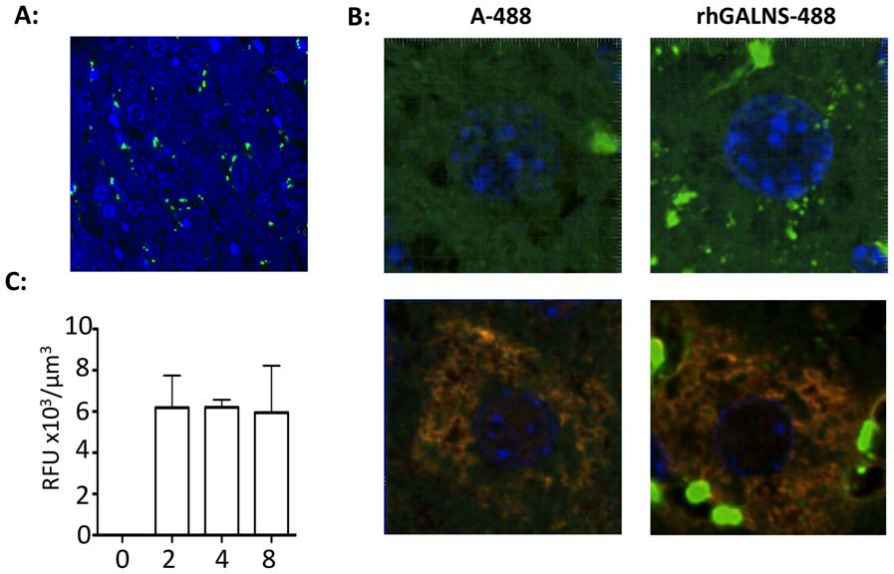
rhGALNS biodistribution in wild-type mouse liver. **A:** Confocal microscopy of direct rhGALNS-A488 fluorescence in liver (green) reveals significant uptake of enzyme into macrophages (Kupffer cells). Blue  =  DAPI nuclear staining. Original magnification 40× **B:** High power confocal microscopy shows uptake of rhGALNS-A488 by hepatocytes by direct fluorescence alone (upper panel) and after staining hepatocytes for albumin (red, lower panel). Original magnification 63× with 5.7× zoom. **C:** Confocal stacks at 40× were analyzed for fluorescence intensity and data presented as average fluorescent signal (RFU)/µm^3^. Measurements of sections from mice that were either injected with A-488/PBS (0), or rhGALNS-A488 and sacrificed at 2 hr (2), 4 hr (4), or 8 hr (8) after injection (n = 3). Means ±SEM are shown.

MPS IVA patients experience accumulation of skeletal KS in the liver [41], [42] and some experience hepatomegaly [43]. Our studies revealed significant rhGALNS-A488 delivery in the sinusoidal cells and macrophages of the liver (Kuppfer cells) (**Figure 7A**). Correction of enzyme levels and clearance of KS accumulation in macrophages may have important clinical implications, as these cells are significantly affected in multiple MPS disorders [44], including MPS IVA [4], [5], and as such are contributing to inflammation and tissue dysfunction. In another MPS disorder, Gaucher disease, ERT resulted in increased macrophage function leading to hematologic and splenohepatic improvements [45]. Confocal microscopy at high magnification revealed enzyme uptake by albumin-positive hepatocytes (**Figure 7B**). Enzyme uptake in liver was higher than other tissues examined (**Figure 7C**).

### Summary

rhGALNS has the potential to reach clinically relevant tissues, including cartilage, heart valve and macrophages, to be taken into lysosomes, by M6P receptors, and to clear accumulated KS, thereby potentially preventing the progression of MPS IVA disease. Amelioration of aberrant gene expression by rhGALNS suggests that enzyme replacement therapy may have an effect on pathophysiology that goes beyond reducing lysosomal storage, and results in restoration of normal cellular physiology.

## Methods

### Mice

Female 4–5 week-old weight-matched Balb/c mice were used for experiments in accordance with the Institutional Animal Care and Use Committee of the Buck Institute for Age Research, protocol number 10150.

### Production and purification of rhGALNS

cDNAs for human GALNS and SUMF-1, each subcloned into a eukaryotic expression vector pCDNA4 containing the Zeocin resistance marker (Invitrogen), were transfected into CHO cells. After establishing a stable pool in Zeocin-containing medium, individual clones were selected by limited dilution. Clones were expanded and adapted to suspension cultures in production medium (Ex-Cell 302, JRH Biosciences). Cell culture fluid containing rhGALNS was filtered, concentrated ∼20-fold and diafiltered into acetate buffer at pH 5.5, pH adjusted and filtered prior to loading onto an ion-exchange column. Protein impurities were removed on an IMAC and a hydrophobic interaction chromatography column. The eluate was concentrated and diafiltered into the formulation buffer. Enzyme purity was ascertained by SDS-PAGE (4–12%) and reverse-phase HPLC methods. Total protein concentrations were determined by Bradford protein assay.

### rhGALNS ELISA and capture activity assay

For quantification of total rhGALNS, molecules were captured by polyclonal GALNS antibodies (BioMarin) and incubated with an rhGALNS antibody conjugated to HRP (BioMarin). Tetramethylbenzidine substrate induced a colorimetric reaction measured at 450 nm. Activity of rhGALNS was determined by modification of a published method [46]. Briefly, rhGALNS molecules, captured by polyclonal GALNS antibodies (5 µg/ml), desulfated 1 mM 4-methylumbelliferyl-galactoside 6-sulphate (4MU-Gal-6S) in 25 mM sodium acetate, pH 4.0 containing 1 mM NaCl (37°C, 30 min). Subsequent reaction with 25 µg/ml β-galactosidase (in 300 mM NaPi pH 7.2, 37°C, 15 min) cleaved the fluorescent molecule 4MU which was quantified by excitation at 355 nm, emission at 460 nm. Amounts of rhGALNS in the samples were extrapolated from a standard curve with known concentrations of rhGALNS. Activity of 1U was defined as production of 1 µmole of 4-MU/min at 37°C and pH 4. Activity was converted to and reported as ng of active enzyme per ml, based on a standard reference preparation of purified rhGALNS with specific activity of 2 U/mg. This result was then normalized per µg of total protein present in the cell lysates.

### Coomassie staining and immunoblotting

Proteins were separated by a reducing SDS-PAGE and stained with Coomassie blue or electroblotted to nitrocellulose membranes for immunoblotting. rhGALNS was detected with anti-rhGALNS antibodies (1∶5,000; BioMarin), and alkaline phosphatase-conjugated secondary antibodies (1∶5,000; Promega). Immunoblots were developed with Western BlueTM substrate (Promega).

### rhGALNS fluorophore labeling

rhGALNS was labeled with Alexa Fluor 488 (A-488; Invitrogen) by maleimide chemistry. The overall charge of labeled rhGALNS was unchanged. Labeling efficiency was calculated by UV/Vis absorbance spectroscopy and was always greater than 90% (1∶1, fluorophore:GALNS).

### rhGALNS uptake experiments *in vitro*

Primary human MPS IVA fibroblasts (GM593 cells; Human Genetic Mutant Cell Repository; Camden, NJ) were maintained in DMEM medium supplemented with 10% fetal bovine serum, 1 mM pyruvate and 2 mM L-glutamine. Normal rabbit synoviocytes (CRL-1832; ATCC) were cultured in Ham's F12 medium, supplemented with 10% fetal bovine serum and antibiotics (Penicillin and Streptomycin). In a modified pulse-chase experiment, confluent cells were incubated with rhGALNS and/or rhGALNS-A488 (4 hr, pulse), followed by rhIduronidase (2 hr, chase). Cells were lysed in M-PER (Pierce). rhGALNS in cell lysates was quantified by GALNS ELISA. For mannose-6-phosphate (M6P) competition experiment, fibroblasts were incubated in 2.5 nM rhGALNS in the presence of increasing concentrations of M6P (12.5 µM – 0.4 mM).

### Primary human MPS IVA chondrocytes

Primary human chondrocytes isolated from iliac crest biopsies of two patients with MPS IVA were a gift from Dr. Wilcox (Cedars-Sinai Medical Center, Los Angeles, CA). Unaffected normal human knee chondrocytes were obtained from Lonza (Walkersville, MD). Chondrocyte monolayers were cultured in proliferation medium (CGM, Lonza) and were lysed with M-PER (Pierce) with protease inhibitors (Roche Diagnostics, Manheim, Germany) for determination of rhGALNS activity, or were fixed with 4% paraformaldehyde for immunocytochemistry. Alginate suspension cultures were established [31] and maintained in differentiation medium (CDM, Lonza) and ascorbic acid (100 µg/ml). Weekly samples of alginate beads were taken for histology, immunocytochemistry and estimation of proliferation. For immunocytochemistry, beads were depolymerized in 55 mM Sodium Citrate (Sigma), trypsinized, adhered to salinized slides (Sigma) by centrifugation (2500 RMP, 20 min) and fixed in acetone (5 min). Upon completion of the study, the beads were depolymerized and pelleted as above, then lysed in M-PER with protease inhibitors (Sigma). Total protein content was quantified (NanoDrop).

### Extracellular matrix staining

Beads were fixed in 4% PFA, dehydrated, embedded in paraffin and sectioned (5 µm). Sections were stained in Alcian blue (30 min) and counterstained in hematoxylin for brighfield microscopy.

### Immunocytochemistry

Cells were permeabilized (0.25% Triton/PBS; 5 min) and blocked (10% Normal Goat Serum; 30 min). Primary antibodies, polyclonal anti-GALNS (1 µg/ml), polyclonal anti-GALNS covalently conjugated with Alexa-488 fluorophore, monoclonal anti-KS (1∶200; Chemicon International) and monoclonal or polyclonal anti-LAMP1 antibodies (1∶200; Santa Cruz Biotechnology) were applied 30 min at RT or ON at 4°C. Goat secondary antibodies (1∶200; Invitrogen) were applied for 30 min. In case of synoviocytes this step was omitted. Slides were mounted in ProlongGold with DAPI (Invitrogen).

### Quantitative RT-PCR (qPCR)

Chondrocyte RNA was extracted (RNeasy Plus Mini Kit, Qiagen) and used to generate cDNAs (High capacity RNA-to-cDNA kit; Applied Biosystems). Gene expression was quantified using probe-based TaqMan q-PCR assays (Applied Biosystems) and the LightCycler 480 Real-Time PCR System (Roche). A crossing point (CP) determined for each gene of interest, using a Second Derivative Maximum Method, was normalized to the mean CP for Glyceraldehyde 3-phosphate dehydrogenase (GAPDH) in the same sample.

### Laser Induced Fluorescence-Capillary Zone Electrophoresis (CE)

Cell lysates were digested with keratanase II, producing disaccharides from KS. The disaccharides were derivatized by reductive amination with the fluorescent dye 2-Aminoacridone, and measured by the P/ACE MDQ CE (BeckmanCoulter) using Laser Induced Fluorescence (LIF), with the laser excitation wavelength at 488 nm. The assay measured KS independently of its molecular size or processing state.

### Biodistribution of rhGALNS-A488

Five bolus administrations of 10 mg/kg rhGALNS-A488 (n = 3/group), PBS/Cys-A488 (n = 2/group) or PBS (n = 1/group), were injected in the tail vein every two days. Heart, liver, femurs and tibias were dissected 2 hr (rhGALNS-A488, PBS/Cys-A488, PBS), 4 hr (rhGALNS-A488) and 8 hr (rhGALNS-A488, PBS/Cys-A488, PBS) after the last treatment. Tissues were fixed in 4% PFA, dehydrated, paraffin embedded and sectioned at 7 µM. Bones were decalcified in 10% Formic Acid/PBS until no calcium oxalate precipitate formed with 5% ammonium oxalate. Sections were deparaffinized and rehydrated prior to antigen retrieval in 10 mM citrate (30 min, 80°C), then blocked (1% normal donkey serum, 0.1% bovine serum albumin, 0.1% NaN_3_, 0.3% Triton X-100 in PBS; 1 hr; RT) and incubated in polyclonal GALNS antibodies, polyclonal mouse albumin antibodies (Abcam) or monoclonal LAMP-1 antibody (4°C, ON). Secondary donkey anti-mouse or rabbit antibodies, conjugated to Alexa-555 were applied (1 hr, RT; Invitrogen). Sections were mounted in ProLong Gold with DAPI. For quantification of signal intensity, confocal stacks were acquired using a Zeiss LSM 510 NLO with a 40× objective, 2× zoom and 0.53 µm z increment.

## Supporting Information

Figure S1 rhGALNS characterization. A: GALNS exhibited affinity to hydroxyapatite, comparable to osteopontin (R&D systems) and arylsulfatase B (ASB; BioMarin). α- glucosidase (BioMarin) exhibited no affinity to hydroxyapatite. Affinities to 100 µg hydroxyapatite, in the presence of 50 µg/ml of BSA were determined by HPLC. B: The oligosaccharide profile of GALNS was generated by PNGF digestion, followed by APTS labeling and CE. Oligosaccharide peaks 1-5 are phosphorylated oligomannose and constitute 50% of the total profile.
(0.28 MB TIF)

Figure S2 Chondrocyte proliferation. Cells released from alginate cultures were permeabilized in 0.25% Triton/PBS (5 min) and stained with DAPI (Invitrogen). Nuclei of 45 cell aggregates (≥3 cells) were counted. Cell numbers were quantified by counting nuclei in 45 cell aggregates per sample. Mean cell numbers ±SEM per cell aggregate are shown. Photomicrographs of an individual clonal aggregate of unaffected and MPS IVA cells in alginate cultures are shown.
(0.34 MB TIF)

Figure S3 GALNS uptake by chondrocytes in alginate. A: MPS IVA chondrocytes were grown for 6 weeks in the presence of 1 nM and 10 nM rhGALNS. B: MPS IVA chondrocytes were grown for 6 weeks, then incubated with 10 nM rhGALNS for additional 9 weeks. GALNS =  green, LAMP1 =  red). All images were acquired with identical parameters.
(5.32 MB TIF)

Figure S4 KS immunofluorescence in MPS IVA chondrocytes from patient 1. Cells were incubated with 10 nM rhGALNS for 6 weeks (terminal time point shown) in alginate suspension cultures. Images were acquired with identical parameters. KS  =  green, LAMP1  =  red. Arrowheads  =  extracellular KS; Arrows  =  intracellular KS.
(4.95 MB TIF)

Figure S5 Capillary electrophoresis (CE) of keratanase II-digested MPS IVA chondrocyte lysates. MPS IVA chondrocytes were grown for 6 weeks, then incubated with 10 nM rhGALNS for additional 9 weeks. Green  =  MPS IVA chondrocytes; Yellow =  MPS IVA chondrocytes treated with 1 nM rhGALNS; Red  =  bovine corneal KS standard. Arrows: disaccharide peaks Gal6S-GlcNAc6S and Gal-GlcNAc6S. Asterisk: GlcNAc6S internal control.
(0.46 MB TIF)

Figure S6 GALNS uptake and KS immunofluorescence in unaffected chondrocytes. Unaffected chondrocytes were grown for 6 weeks in the presence 10 nM rhGALNS in alginate suspension cultures. Images were acquired with identical parameters. A: GALNS  =  green, LAMP1  =  red. B: KS  =  green, LAMP1  =  red.
(2.85 MB TIF)

Figure S7 Visualization of rhGALNS. A: Rabbit synoviocytes exhibit comparable uptake of rhGALNS and rhGALNS-A488 by GALNS ELISA. B: Comparison of direct (rhGALNS-A488, green, left panel) and amplified indirect (anti-GALNS antibodies, secondary antibodies conjugated to A555, red, right panel) detection of GALNS in rabbit synoviocytes treated with rhGALNS (10 nM, 4 hrs). Arrows show examples of points of equivalent signal distribution.
(0.71 MB TIF)
