# Supplementary figures and images for: Enzyme Replacement in a Human Model of Mucopolysaccharidosis IVA In Vitro and Its Biodistribution in the Cartilage of Wild Type Mice
Source: PLoS One. 2010 Aug 16;5(8):e12194. doi: 10.1371/journal.pone.0012194 (PMC2922370; doi:10.1371/journal.pone.0012194)

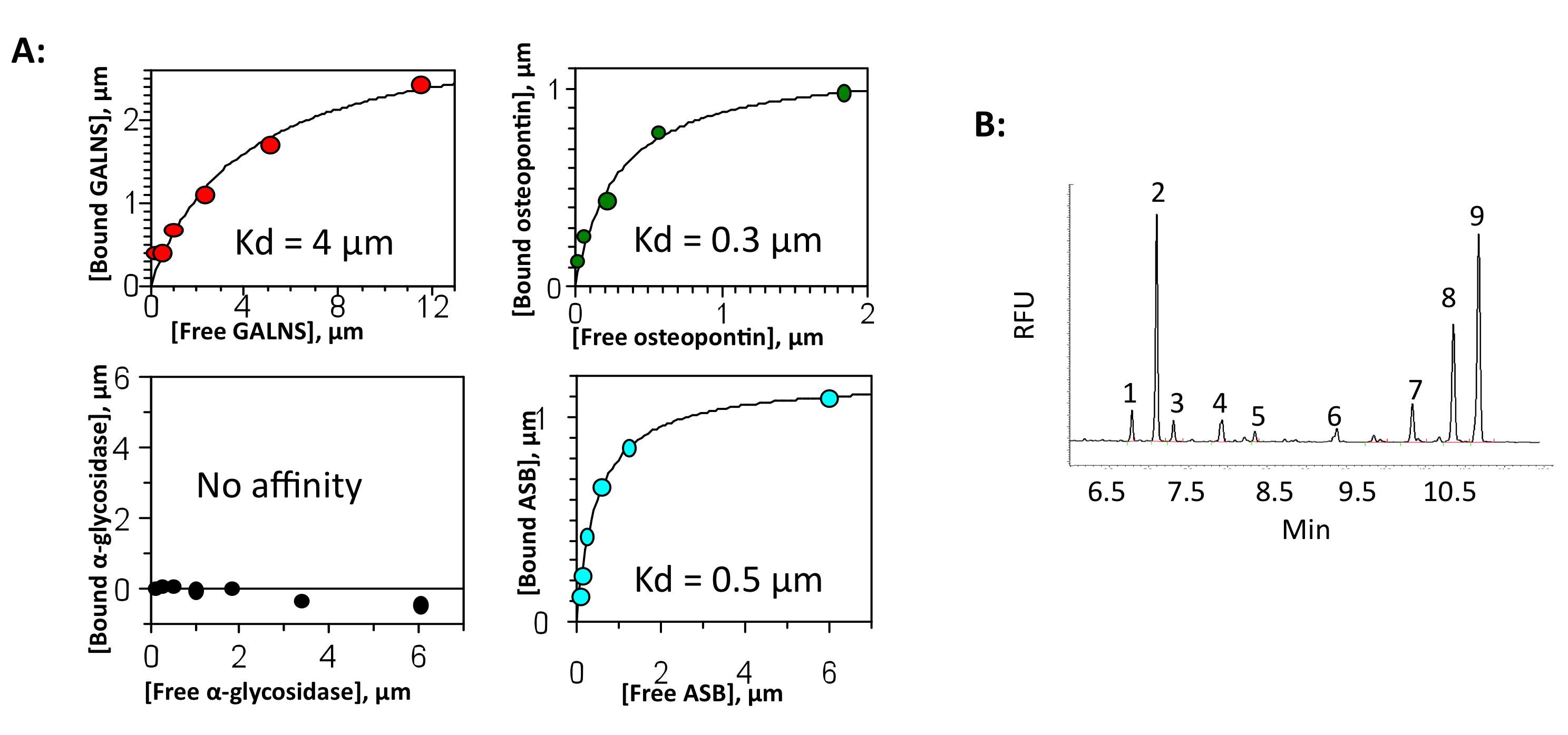

Supplement: Figure S1 — rhGALNS characterization. A: GALNS exhibited affinity to hydroxyapatite, comparable to osteopontin (R&D systems) and arylsulfatase B (ASB; BioMarin). α- glucosidase (BioMarin) exhibited no affinity to hydroxyapatite. Affinities to 100 µg hydroxyapatite, in the presence of 50 µg/ml of BSA were determined by HPLC. B: The oligosaccharide profile of GALNS was generated by PNGF digestion, followed by APTS labeling and CE. Oligosaccharide peaks 1-5 are phosphorylated oligomannose and constitute 50% of the total profile. (0.28 MB TIF) [file pone.0012194.s001.tif]

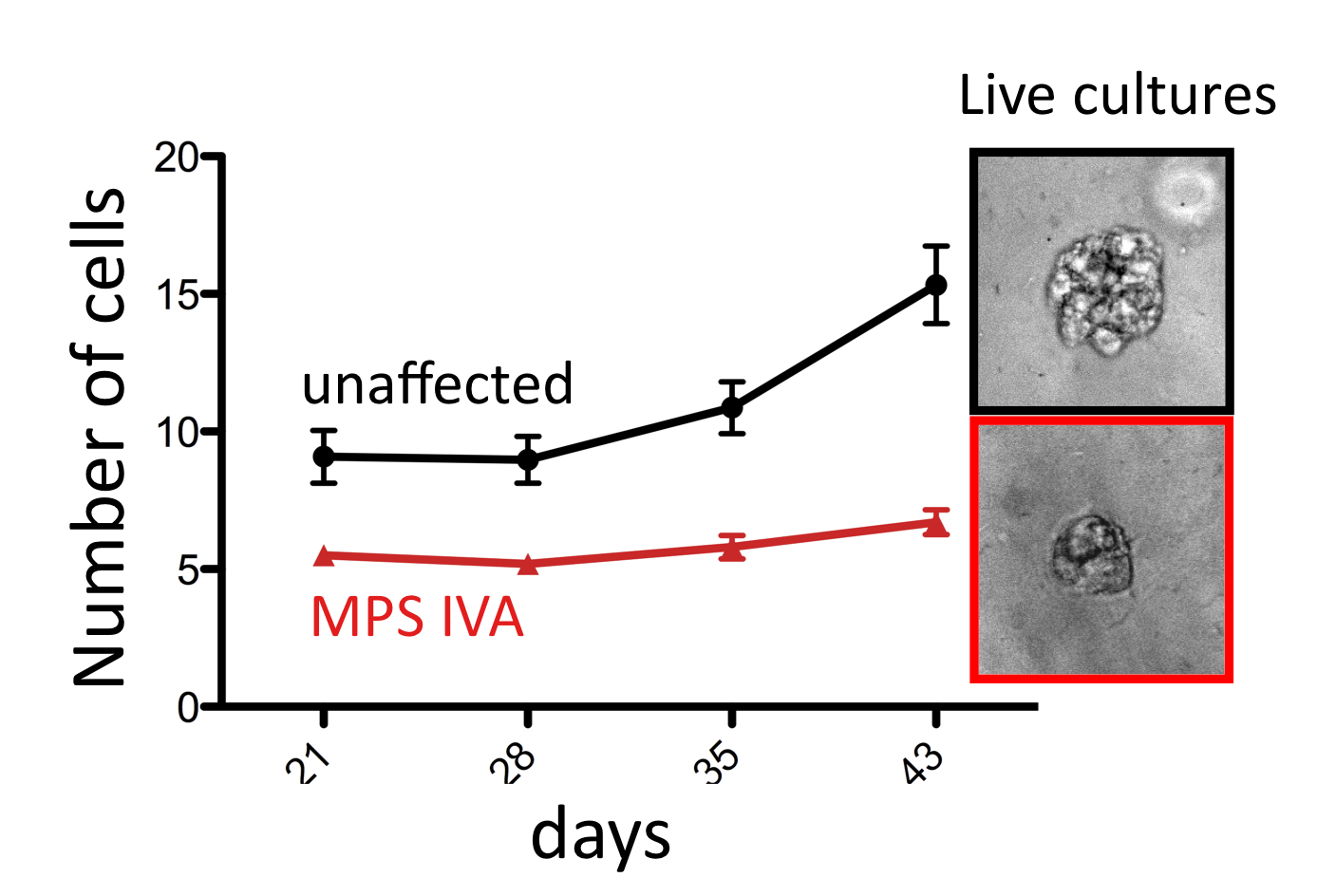

Supplement: Figure S2 — Chondrocyte proliferation. Cells released from alginate cultures were permeabilized in 0.25% Triton/PBS (5 min) and stained with DAPI (Invitrogen). Nuclei of 45 cell aggregates (≥3 cells) were counted. Cell numbers were quantified by counting nuclei in 45 cell aggregates per sample. Mean cell numbers ±SEM per cell aggregate are shown. Photomicrographs of an individual clonal aggregate of unaffected and MPS IVA cells in alginate cultures are shown. (0.34 MB TIF) [file pone.0012194.s002.tif]

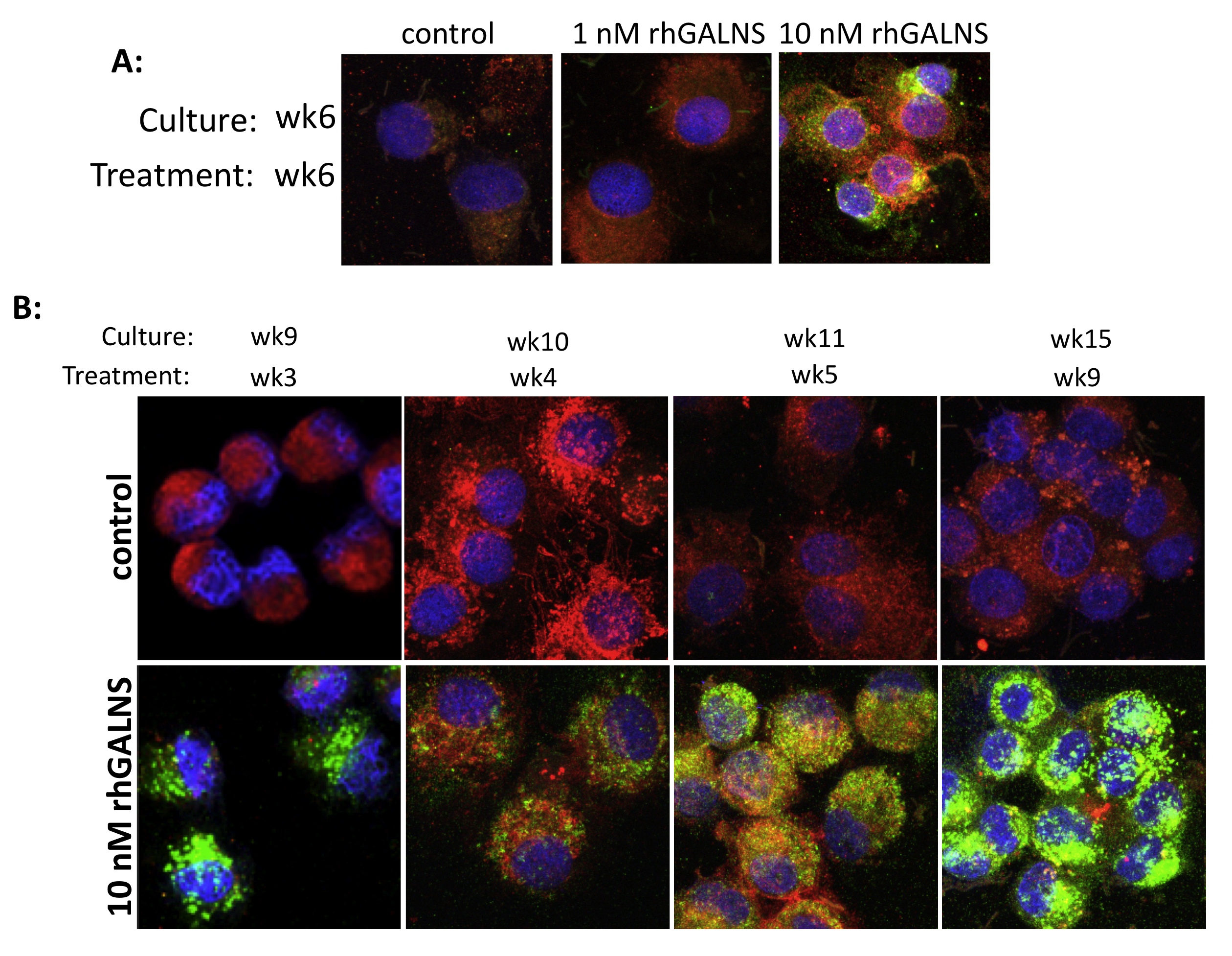

Supplement: Figure S3 — GALNS uptake by chondrocytes in alginate. A: MPS IVA chondrocytes were grown for 6 weeks in the presence of 1 nM and 10 nM rhGALNS. B: MPS IVA chondrocytes were grown for 6 weeks, then incubated with 10 nM rhGALNS for additional 9 weeks. GALNS = green, LAMP1 = red). All images were acquired with identical parameters. (5.32 MB TIF) [file pone.0012194.s003.tif]

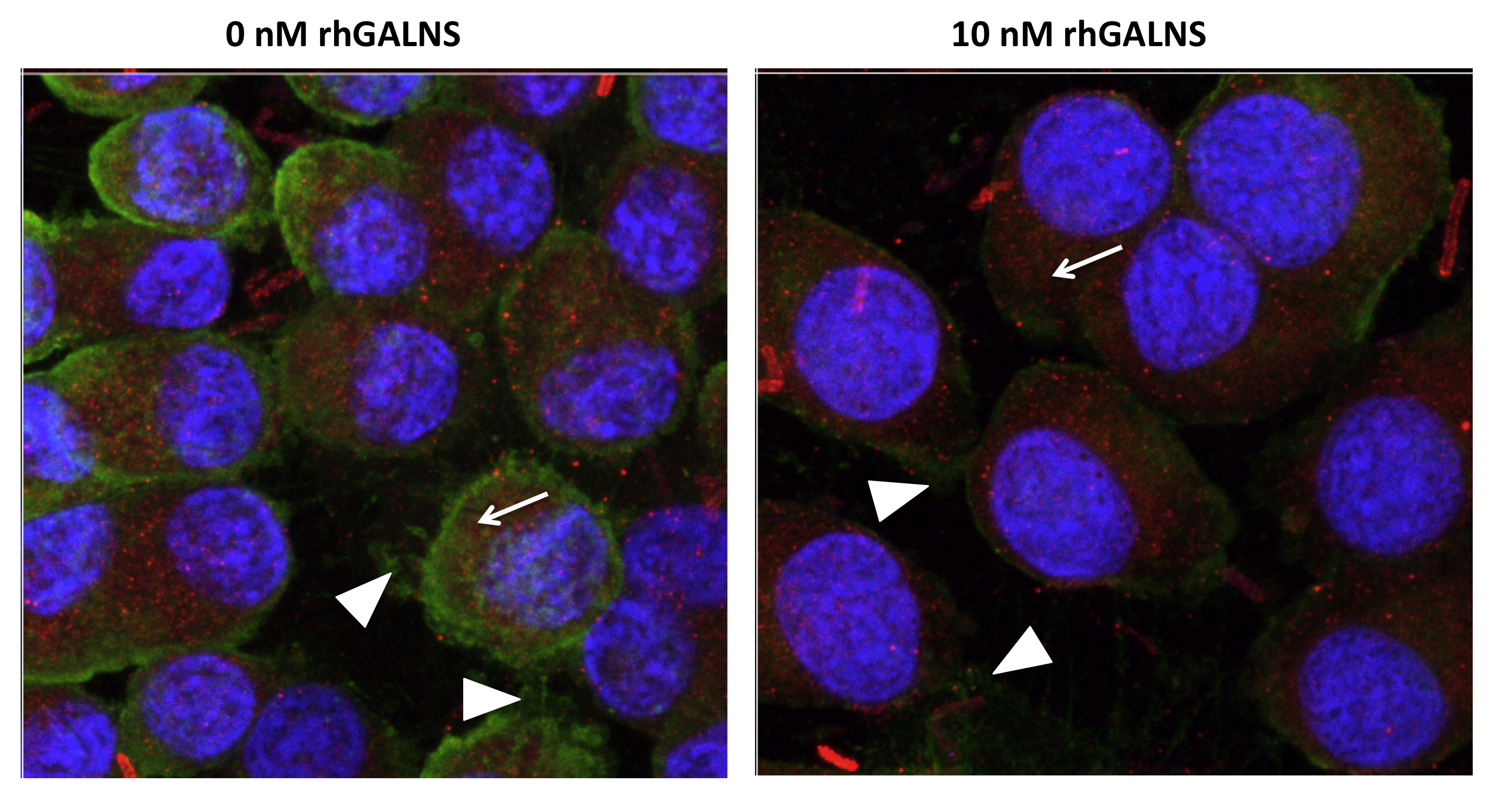

Supplement: Figure S4 — KS immunofluorescence in MPS IVA chondrocytes from patient 1. Cells were incubated with 10 nM rhGALNS for 6 weeks (terminal time point shown) in alginate suspension cultures. Images were acquired with identical parameters. KS = green, LAMP1 = red. Arrowheads = extracellular KS; Arrows = intracellular KS. (4.95 MB TIF) [file pone.0012194.s004.tif]

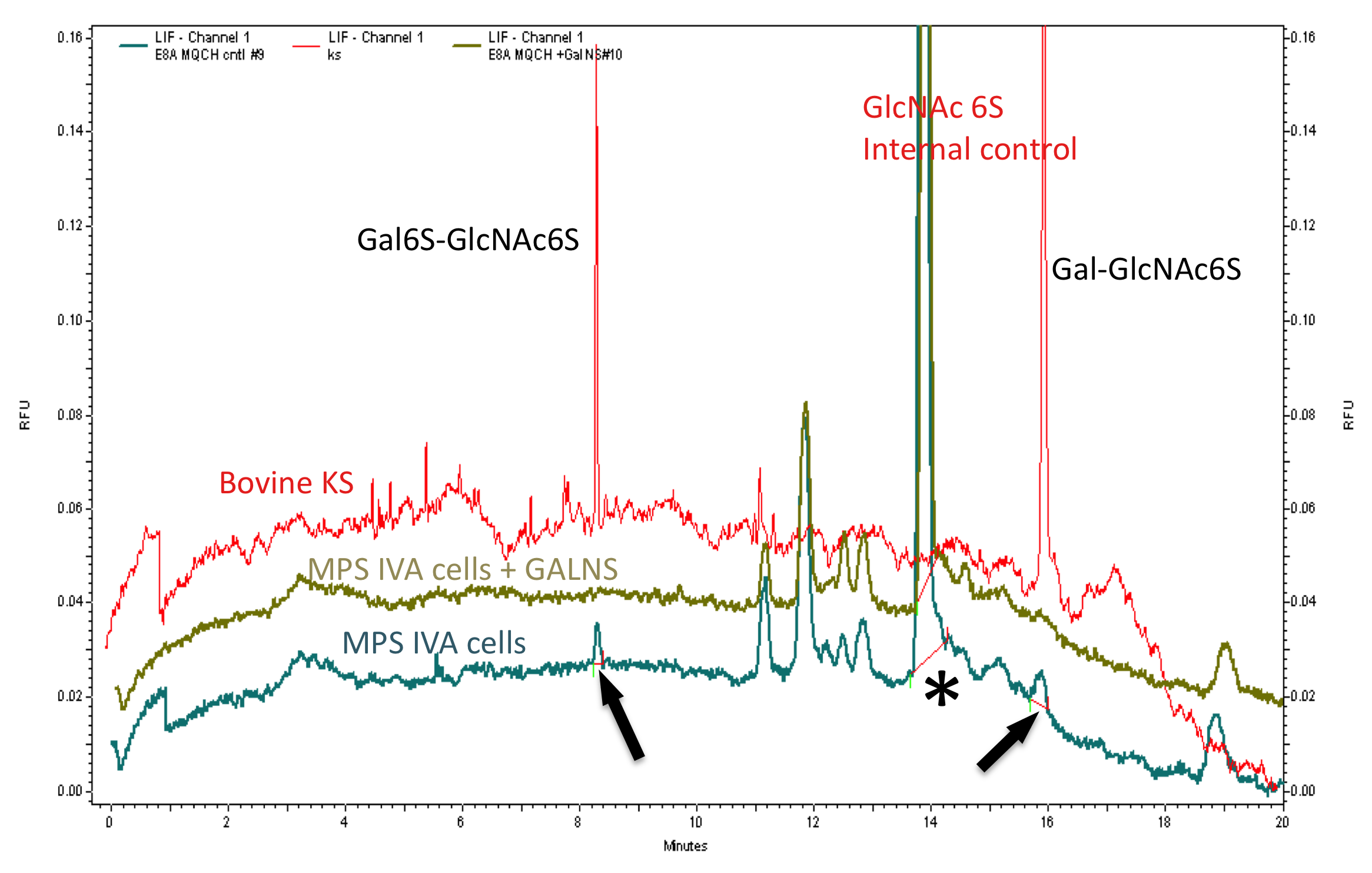

Supplement: Figure S5 — Capillary electrophoresis (CE) of keratanase II-digested MPS IVA chondrocyte lysates. MPS IVA chondrocytes were grown for 6 weeks, then incubated with 10 nM rhGALNS for additional 9 weeks. Green = MPS IVA chondrocytes; Yellow = MPS IVA chondrocytes treated with 1 nM rhGALNS; Red = bovine corneal KS standard. Arrows: disaccharide peaks Gal6S-GlcNAc6S and Gal-GlcNAc6S. Asterisk: GlcNAc6S internal control. (0.46 MB TIF) [file pone.0012194.s005.tif]

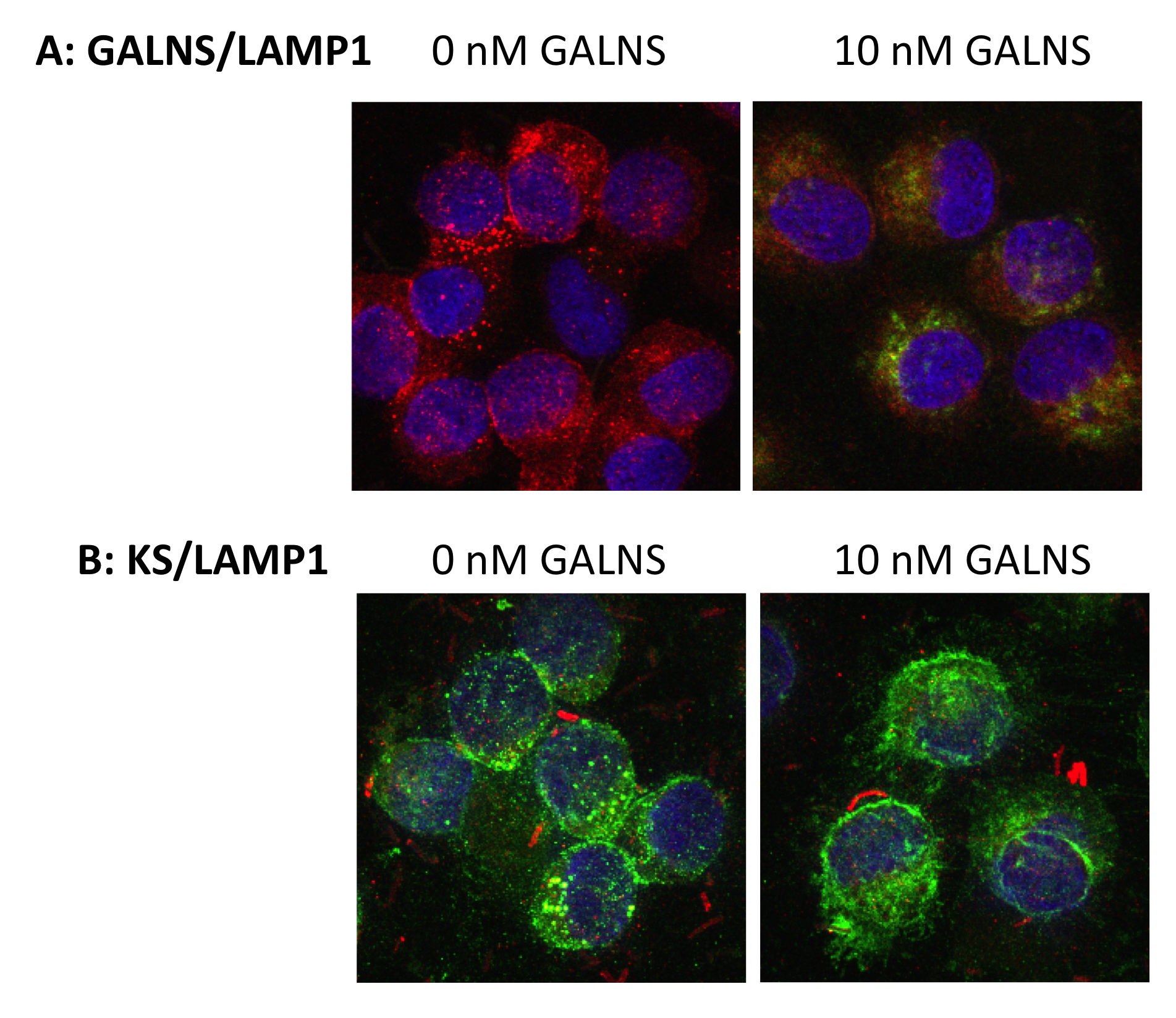

Supplement: Figure S6 — GALNS uptake and KS immunofluorescence in unaffected chondrocytes. Unaffected chondrocytes were grown for 6 weeks in the presence 10 nM rhGALNS in alginate suspension cultures. Images were acquired with identical parameters. A: GALNS = green, LAMP1 = red. B: KS = green, LAMP1 = red. (2.85 MB TIF) [file pone.0012194.s006.tif]

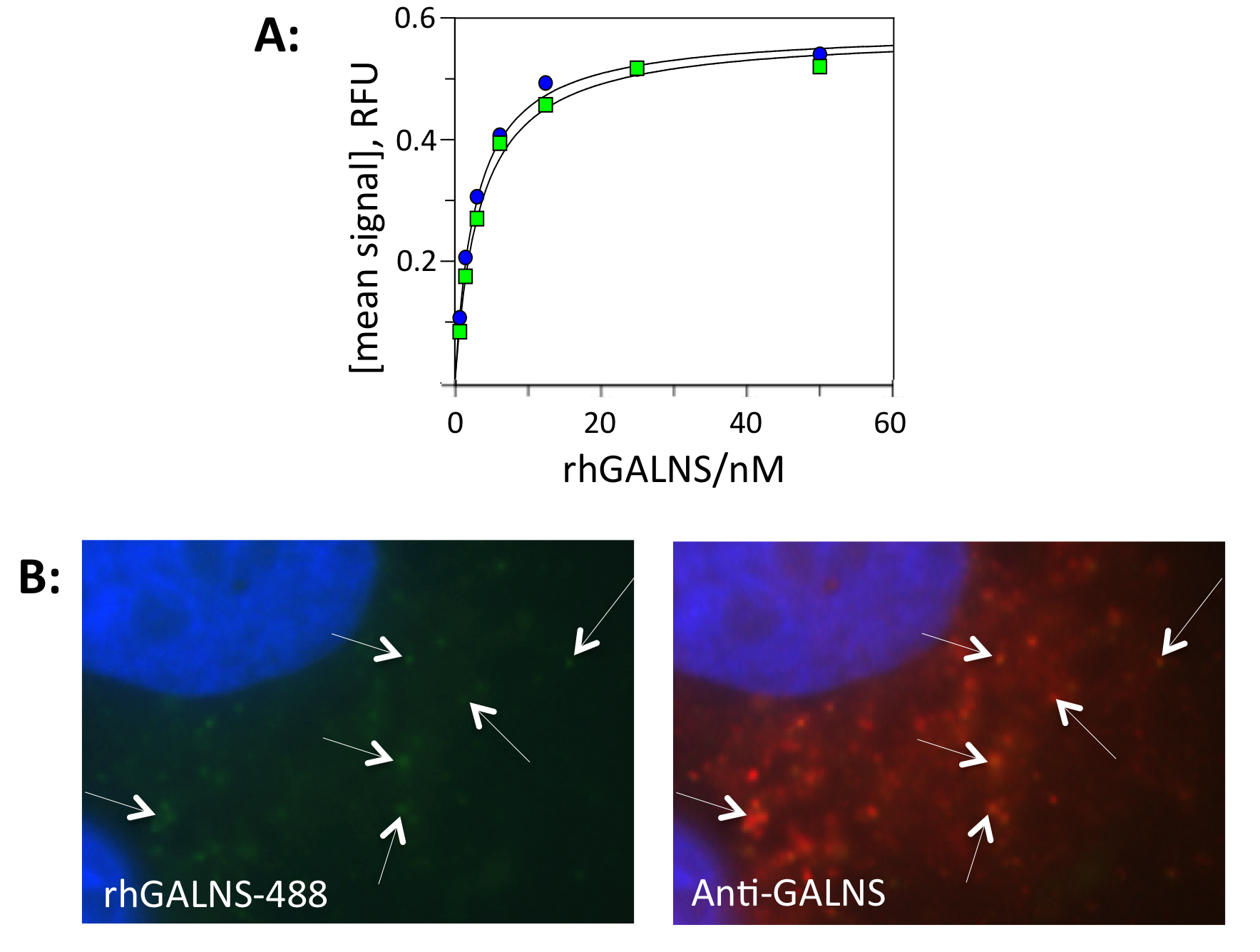

Supplement: Figure S7 — Visualization of rhGALNS. A: Rabbit synoviocytes exhibit comparable uptake of rhGALNS and rhGALNS-A488 by GALNS ELISA. B: Comparison of direct (rhGALNS-A488, green, left panel) and amplified indirect (anti-GALNS antibodies, secondary antibodies conjugated to A555, red, right panel) detection of GALNS in rabbit synoviocytes treated with rhGALNS (10 nM, 4 hrs). Arrows show examples of points of equivalent signal distribution. (0.71 MB TIF) [file pone.0012194.s007.tif]
